# Case-control study of oral disease indexes in individuals with head and neck cancer after antineoplastic therapy

**DOI:** 10.1590/S1679-45082018AO4245

**Published:** 2018-08-01

**Authors:** Reyna Aguilar Quispe, Adrielle Lindolpho Cremonesi, Jeanne Kelly Gonçalves, Cassia Maria Fischer Rubira, Paulo Sérgio da Silva Santos

**Affiliations:** 1 Faculdade de Odontologia de Bauru, Universidade de São Paulo, Bauru, SP, Brazil.

**Keywords:** Dental caries, Periodontal diseases, Mouth rehabilitation, Head and neck neoplasms/drug therapy, Radiotherapy, Cárie dentária, Doenças periodontais, Reabilitação bucal, Neoplasias de cabeça e pescoço/tratamento farmacológico, Radioterapia

## Abstract

**Objective:**

To evaluate the oral health of patients with head and neck cancer after antineoplastic treatment, and to compare them with patients with no history of cancer.

**Methods:**

A total of 75 patients, divided into Study Group, composed of individuals after antineoplastic treatment (n=30), and Control Group, with individuals with no history of cancer (n=45), aged 37 to 79 years. The oral health status was evaluated through the index of decayed, missing or filled permanent teeth (DMFT), community periodontal index and evaluation of the use and need of prosthesis. All of these items were evaluated according to the criteria recommended by the World Health Organization. The statistical analysis was descriptive and used the Pearson’s χ^2^ test.

**Results:**

The community periodontal index was higher in the Study Group when compared to the Control Group (p<0.0001). The need for an upper (p<0.001) and lower (p<0.0001) prostheses was higher in the Study Group. Also, the use of upper prosthesis was higher in the Study Group (p<0.002). The missing or filled permanent teeth index between the two groups (p>0.0506) and the use of lower prosthesis (p>0.214) did not present a relevant statistical difference.

**Conclusion:**

Periodontal disease and edentulism are the most significant changes in individuals who received antineoplastic therapy for head and neck cancer as well as greater need for oral rehabilitation.

## RESUMO

**Objetivo:**

Avaliar a saúde bucal de pacientes com câncer de cabeça e pescoço após tratamento antineoplásico, e compará-los com pacientes sem histórico de câncer.

**Métodos:**

Foram avaliados 75 pacientes, divididos em Grupo de Estudo de indivíduos após tratamento antineoplásico (n=30) e Grupo de Controle de indivíduos sem histórico de câncer (n=45), com faixa etária de 35 a 79 anos. A condição de saúde oral foi avaliada pelo índice de dentes permanentes cariados, perdidos e obturados (CPOD), pelo índice periodontal comunitário e por uma avaliação de uso e necessidade de prótese, conforme critérios preconizados pela Organização Mundial da Saúde. A análise estatística foi descritiva e realizada por meio do teste do χ^2^ de Pearson.

**Resultados:**

O índice periodontal comunitário foi maior no Grupo de Estudo quando comparado ao controle (p<0,0001). A necessidade de prótese superior (p<0,001) e inferior (p<0,0001) foi maior no Grupo de Estudo. O uso de prótese superior foi maior no Grupo de Estudo (p<0,002). O índice de dentes permanentes cariados, perdidos e obturados entre os dois grupos (p>0,0506) e o uso de prótese inferior (p>0,214) não apresentaram diferença estatística relevante.

**Conclusão:**

A doença periodontal e o edentulismo são as alterações mais significativas em indivíduos que receberam terapia antineoplásica de câncer de cabeça e pescoço, assim como maior necessidade de reabilitação oral.

## INTRODUCTION

It is estimated there will be 1,031,439 new cases per year of head and neck cancer (HNC) worldwide, in 2030.^(^ ^1^ ^)^ The lips, oral cavity, oropharynx, nasopharynx, pharynx, larynx, salivary glands, and nasal and paranasal sinuses are the anatomical structures involved in HNC.^(^ ^2^ ^)^

The antineoplastic treatment (AT) for patients with HNC consists of surgery, chemotherapy, radiotherapy, or combined therapy. These treatments are given according to staging and site of the tumor.^(^ ^2^ ^)^ Side effects may occur with these treatments, and depending on the site, can be local and/or systemic, and according to their duration, are classified as acute or chronic. The type and degree of manifestation of these side effects depend on the type and dose of the AT.^(^ ^3^ ^)^

In the oral cavity, the acute effects of AT include oral mucositis, changes in viscosity and volume of saliva, dysgeusia, candidiasis, and limited movement. The chronic effects include neuropathy, atrophy of the facial muscles and salivary glands, halitosis, dysphagia, dysphonia, osteoradionecrosis, xerostomia, hyposalivation, dental caries, and periodontal disease.^(^ ^3^ ^,^ ^4^ ^)^

Regular follow-up by a multiprofessional team is fundamental for preserving the health of HNC survivors. In the dentistry area, it is essential to perform regular check-ups as a preventive measure against dental caries, periodontal diseases, and possible infectious conditions,^(^ ^5^ ^,^ ^6^ ^)^ especially to decrease the high risk of developing osteoradionecrosis that still exists several years after radiotherapy.^(^ ^7^ ^,^ ^8^ ^)^

Some studies showed that individuals with HNC after the AT presented with a higher prevalence of dental caries and periodontal disease when compared to individuals who did not undergo such treatment.^(^ ^6^ ^,^ ^9^ ^)^ However, data on the oral health of individuals with HNC after AT are still scarce.^(^ ^7^ ^)^

For this reason, as a part of the multiprofessional team that accompanies patients with HNC, dental surgeons have the role of deepening their knowledge regarding oral health conditions after AT, so that they might offer alternatives of treatment and dental maintenance, aiming at better quality of life for this population.^(^ ^4^ ^,^ ^10^ ^)^

## OBJECTIVE

To evaluate the oral health of patients with head and neck cancer after oncologic treatment, and to compare it with that of individuals with no history of antineoplastic treatment.

## METHODS

This is a cross-sectional comparative case-control study conducted at the *Centro de Pesquisa Clínica da Faculdade de Odontologia de Bauru da Universidade de São Paulo,* from August, 2014, to July, 2015, under official opinion no. 703.115, CAAE no. 31088414.5.0000.5417. All research participants were informed about the procedures done and signed the Informed Consent Form.

Individuals from both sexes older than 18 years were evaluated and divided into Study Group (SG) and Control Group (CG). The SG was composed of patients with HNC after the conclusion of treatment, including surgery, chemotherapy, and/or radiotherapy. The information recorded was age, sex, type of treatment received, region of the cancer, and time of conclusion of AT. Excluded were patients with neurological diseases and/or cancer at the time of the clinical examination. The CG comprised patients in good general health and with no history of cancer.

The oral health condition assessment was performed by a single evaluator calibrated by means of the Decayed, Missing, and Filled Teeth (DMFT) index, of the Community Periodontal Index (CPI), and the assessment of need and use of dental prosthesis. For all these evaluations, the criteria recommended by the World Health Organization (WHO) were applied.^(^ ^11^ ^)^

For the statistical analysis of the results, descriptive statistics and Pearson’s χ^2^ test were used to relate the oral health condition with the AT of the SG, and compare it with the CG. The level of significance was set as p<0.05.

## RESULTS

Seventy-five individuals were assessed, 30 in the SG and 45 in the CG, of which 25 were men (83.3%) in the SG and 28 women (62.2%) in the CG. The age range for both groups was 35 to 79 years, with a mean age of 60.27 and 55.76 years, respectively in SG and CG. The most often affected sites in the SG were tongue, tonsils, and larynx. The antineoplastic treatments of the SG were chemotherapy, radiotherapy, and surgery. Among the HNC patients, 15 were evaluated after concluding AT for less than one year, and 15 individuals after one year, and the interval range was 1 month to 9 years (Table 1).

**Table 1:** Distribution of characteristics of individuals with head and neck cancer

Patient	Sex	Age	Type of cancer	Surgery	Radiotherapy	Chemotherapy	Time after treatment
1	Male	35	Nasopharynx	Yes	Yes	No	4 years and 9 months
2	Male	41	Amygdala	No	Yes	Yes	3 months
3	Male	42	Amygdala	Yes	Yes	Yes	7 months
4	Male	45	Amygdala	No	Yes	Yes	1 month
5	Male	46	Tonsillar pillar	No	Yes	Yes	1 year and 1 month
6	Male	49	Hypophysis	No	Yes	Yes	15 years
7	Male	51	Tonsillar fossa	Yes	Yes	Yes	4 months
8	Female	52	Mucoepidermoid	Yes	No	No	2 years and 2 months
9	Male	52	Tongue	No	Yes	Yes	4 months
10	Male	52	Floor of the mouth	No	Yes	Yes	1 year and 4 months
11	Male	55	Tongue	Yes	Yes	No	10 months
12	Male	57	Tongue	No	Yes	Yes	1 year
13	Male	59	Nasopharynx	Yes	Yes	Yes	2 years and 5 months
14	Male	60	Larynx	Yes	Yes	No	1 year
15	Male	61	Larynx	Yes	Yes	No	9 years and 5 months
16	Male	61	Gingiva	Yes	No	No	3 months
17	Male	62	Vocal folds	Yes	Yes	No	2 months
18	Male	63	Amygdala	Yes	Yes	No	1 year and 11 months
19	Male	64	Tongue	Yes	Yes	Yes	1 year and 5 months
20	Male	65	Floor of the mouth	Yes	Yes	No	2 years
21	Female	65	Adenoid	Yes	Yes	No	9 months
22	Female	69	Osteosarcoma	Yes	Yes	No	2 years and 9 months
23	Male	70	Tongue	Yes	Yes	No	6 months
24	Female	72	Gingiva	Yes	Yes	Yes	2 months
25	Male	73	Tongue	No	Yes	Yes	1 month
26	Male	74	Lip	Yes	No	Yes	1 year and 2 months
27	Female	77	Tongue mucosa	Yes	Yes	Yes	5 months
28	Male	78	Larynx	No	Yes	Yes	4 months
29	Male	79	Tonsillar pillar	Yes	Yes	Yes	7 months
30	Male	79	Vocal folds	Yes	Yes	No	7 years

As to the DMFT, the mean was 24.43 for the SG and 25.24 for the CG, with no statistically significant difference (p>0.506). The CPI revealed a higher prevalence of periodontal disease in the SG (96.7%) compared to the CG (60%). The SG presented with a greater presence of calculi (33.3%), followed by shallow periodontal pocket (26.7%) compared to CG (40%), which presented with a higher number of individuals with no clinical signs of periodontal disease (p<0.0001) (Table 2).

**Table 2:** Assessment of periodontal condition as per the community periodontal index

Criteria	SG	CG
	n (%)	n (%)
Healthy	1 (3.3)	18 (40)
Bleeding observed, directly or by using a mouth mirror, after probing	4 (13.3)	8 (8)
Calculus (any amount)	10 (33.3)	10 (22.2)
4 to 5mm pocket	8 (26.7)	0 (0)
6mm or larger pocket	1 (3.3)	0 (0)
When less than two functional teeth are present	3 (10)	9 (20)
Excluded sextant	3 (10)	0 (0)
Total	30 (100)	45 (100)
Pearson’s χ^2^ test	30.863	
p value*	<0.0001	

* p value - significance level p<0.005.

SG: Study Group; CG: Control Group.

The use of an upper dental prosthesis was greater in the SG (60%) in comparison with the CG (13.7%), with a statistically significant difference (p<0.002). Nevertheless, both groups used some type of lower dental prosthesis (99.9%) with no statistically significant difference (p>0.214) (Table 3).

**Table 3:** Assessment of use of upper and lower prosthesis

Criteria	Upper		Lower	
	
	SG	CG	SG	CG
	n (%)	n (%)	n (%)	n (%)
Does not use dental prosthesis	12 (40)	39 (86.7)	24 (80)	41 (91.1)
Uses a fixed bridge	4 (13.3)	2 (4.4)	1 (3.3)	0 (0)
Uses more than one fixed bridge	6 (20)	1 (2.2)	4 (13.3)	1 (2.2)
Uses a removable partial denture	1 (3.3)	1 (2.2)	1 (3.3)	2 (4.4)
Uses one or more fixed bridges and one removable partial denture	1 (3.3)	0 (0)	0 (0)	0 (0)
Uses a complete denture prosthesis	6 (20)	2 (4.4)	0 (0)	1 (2.2)
No information	0 (0)	0 (0)	0 (0)	0 (0)
Total	30 (100)	45 (100)	30 (100)	45 (100)
Pearson’s χ^2^ test	19.304		5.812	
p value*	<0.002		<0.214	

*p value - significance level p<0.005.

SG: Study Group; CG: Control Group.

Individuals from the SG (70%) presented with a greater need for an upper dental prosthesis than those of the CG (22.2%), with a significant difference (p<0.001). Also, the SG (96.7%) showed a notable need for a lower dental prosthesis when compared to the CG (22.2%), with a statistically significant difference (p<0.0001) (Table 4).

**Table 4:** Assessment of need for upper and lower dental prosthesis

Criteria	Upper		Lower	
	
	SG	CG	SG	CG
	n (%)	n (%)	n (%)	n (%)
Does not need dental prosthesis	9 (30)	35 (77.8)	1 (3.3)	35 (77.8)
Needs a dental prosthesis to replace one element	3 (10)	2 (4.4)	0 (0)	4 (8.9)
Needs a dental prosthesis to replace more than one element	10 (33.3)	3 (6.7)	21 (70)	2 (4.4)
Needs a combination of dental prosthesis	4 (13.3)	1 (2.2)	1 (3.3)	1 (2.2)
Needs a complete denture prosthesis	4 (13.7)	3 (6.7)	7 (23.3)	3 (6.7)
No information	0 (0)	1 (2.2)	0 (0)	0 (0)
Total	30 (100)	45 (100)	30 (100)	45 (100)
Pearson’s χ^2^ test	20.079		52.507	
p value*	<0.001		<0.0001	

*p value - significance level p<0.005.

SG: Study Group; CG: Control Group.

## DISCUSSION

There are several predisposing factors to developing HNC. The most frequent among them are consumption of alcohol and tobacco. In the oral cavity, they cause changes in saliva, allowing a greater colonization of different strains of *Candida* in individuals with greater risk of oral cancer.^(^ ^12^ ^)^ These poor habits, when associated with deficient oral hygiene, increase even further the risk of developing cancer, especially in the mouth.^(^ ^13^ ^,^ ^14^ ^)^ The socioeconomic factor can also influence in the development of cancer, and 20 million new cases are foreseen for 2025, which should primarily affect the low-income countries.^(^ ^1^ ^)^ This scenario, associated with a lower schooling level of the population, leads to ignoring the importance of performing oral hygiene as a preventive measure against cancer.^(^ ^7^ ^)^

The instructions offered by the dentist to individuals with HNC as to oral health before, during, and after AT can prevent side effects, such as periodontal disease and dental caries.^(^ ^11^ ^,^ ^12^ ^)^ However, the prevalence of these diseases also depends on other associated factors, such as schooling level, socioeconomic factors, and ease of access to dental care.^(^ ^7^ ^,^ ^9^ ^,^ ^15^ ^,^ ^16^ ^)^ In this study, there was a statistically significant difference in the prevalence of dental caries between SG and CG, presenting with a mean DMFT index of 24.43 and 25.24, respectively. Nonetheless, periodontal disease was the most prevalent in the SG, with 90.7% as compared to 60% in the CG. Periodontal tissues can suffer changes after AT, including gingival recessing, loss of gingival insertion, and a high index of bacterial dental plaque.^(^ ^17^ ^)^ A study carried out in Austria revealed that of the total number of individuals with HNC who concluded the AT, one third presented with dental caries, with a mean DMFT of 25.3, and two thirds of them had some sign of periodontitis. It was evident that the greatest problem of these individuals was periodontal disease when compared to dental caries. Patients who presented with a higher prevalence of both diseases were the individuals with a low socioeconomic status and no access to healthcare insurance plans.^(^ ^7^ ^)^

A study that evaluated individuals with HNC when initiating and after concluding AT showed that only 11% of the total number of patients assessed (n=109) had caries. According to the authors, the reduced number of patients with caries might have resulted from the instructions as to care and maintenance of oral health, prescription of fluoride, and frequent check-ups conducted from beginning to end of the AT. They also mentioned the fact of the evaluation having been done soon after conclusion of the AT (4 months) may have interfered in the results, since both caries and periodontal disease evolve chronically.^(^ ^18^ ^)^

In this study, the SG was assessed with a minimum time of 1 month and maximum of 9 years and 5 months after concluding the AT, and 50% of individuals were evaluated at a time shorter than 12 months after AT, which could have interfered in the results of the DMFT index. A study performed in China reported that the post-AT HNC individuals presented with a low CPI when compared to the CG, and stated radiation therapy did not cause periodontitis, but rather, gingival recession. It is important to remember that the CPI does not rate this variable, and it is recommended to use another type of assessment to verify the degree of periodontal insertion.^(^ ^9^ ^)^

A study conducted in India made periodontal assessment at three time points (start of AT, 10 days after AT, and 180 days after AT), and there was no statistically significant difference among the groups. There was good periodontal health in the three groups, since all patients had dental instructions and follow-up from the beginning to the end of AT.^(^ ^19^ ^)^

The patients recruited for this research were referred from several public and private organizations to the outpatient clinic of our institution, and instruction as to oral health care may have varied from one patient to another (such information was not recorded).

The AT may cause modifications in the micromorphology of the dental tissues, such as enamel and dentin.^(^ ^15^ ^)^ When radiation caries are present, the cervical region is the most affected.^(^ ^20^ ^)^ This was evident in a study that evaluated teeth of patients irradiated with 50 Gy to 70 Gy. The teeth were assessed in the regions of the cusps, and occlusal and cervical surfaces, by means of a polarized microscope. It was apparent that the cervical area of the enamel was modified, showing dark areas as well as a larger interprismatic space.^(^ ^20^ ^)^ The dentin can also suffer changes, such as obturation of the dentinal tubules and dehydration, increasing the possibility of caries in this region.^(^ ^21^ ^)^ This study evaluated the prevalence of dental caries according to the DMFT index, which does not allow the record of the specific region of dental caries, for example, the cervical area of a tooth. Consequently, despite this area being the most affected, as per the literature, it was not possible to determine which area of the tooth was affected by the caries.

Between chemotherapy and radiotherapy, the latter affects more the dental structures and periodontal tissues. High doses of radiation and early age of treatment are factors that predispose towards greater damage in these tissues. Further, radiotherapy has chronic effects that may be often irreversible.^(^ ^5^ ^,^ ^20^ ^)^ In this study, 90% of individuals assessed received radiation therapy as treatment.

The lack of dental prostheses in individuals with HNC can hinder functions, such as chewing and phonation, and can have esthetic repercussions, interfering in quality of life in the physical and psychological realms.^(^ ^22^ ^,^ ^23^ ^)^ In this study, the use of an upper dental prosthesis was greater in the SG (60%) as compared to the CG (13.7%), and the need for use of a prosthesis, both upper (p<0.002) and lower (p<0.0001) was greater in the SG than in the CG. In a study of 272 individuals with HNC after AT, 91.8% were edentulous; 30.6% of them were totally edentulous. Approximately half of these patients, when using some type of prosthesis, used only the upper prosthesis − for the esthetic need more than for a functional need. The other half of patients did not use any type of prosthesis. Once rehabilitated with dental prostheses, these patients increased their ingestion of solid food from 40 to 60%.

In this same study, patients were not able to use prosthesis immediately after AT, due to different existing side effects, such as mucositis, edema, and/or xerostomia.^(^ ^24^ ^)^ In the present study, the SG had a greater need for lower prosthesis (96.7%) when compared to the upper prosthesis (30%). This can be explained by the fact that many patients should not and/or are unable to use the lower prosthesis because the mucous membranes, such as the tongue, do not tolerated the use of the prosthesis.^(^ ^25^ ^)^

In a study of 72 individuals with HNC after AT evaluated for the manufacture of the prosthesis, 48 of them presented with a greater necessity and/or problems with the lower prosthesis. AT can cause changes in hard and soft tissues, compromising the stability and retention of the dental prosthesis, and making the rehabilitation of the patients difficult.^(^ ^23^ ^)^ The evaluation of the use and of the need for prosthesis should not only anticipate rehabilitation, by means of the preparation of the prosthesis and/or implants, but also consider that the prosthesis has a large impact on patients’ quality of life, in the physical aspect, such as nutrition, and – primarily - in the psychological aspect, by means of esthetic rehabilitation, allowing social insertion of this population.^(^ ^22^ ^,^ ^23^ ^)^

## CONCLUSION

The oral health of individuals with head and neck cancer is affected after antineoplastic treatment. Among the dental and periodontal structures, the latter present with the highest damage after antineoplastic treatment. The need for use of prosthesis is greater in this population. Dental follow-up after antineoplastic treatment, encouraging and informing patients about oral healthcare, is fundamental for improving quality of life.

## References

[1] Ferlay J, Soerjomataram I, Dikshit R, Eser S, Mathers C, Rebelo M (2015). Cancer incidence and mortality worldwide: sources, methods and major patterns in GLOBOCAN 2012. Int J Cancer.

[2] Cohen EE, LaMonte SJ, Erb NL, Beckman KL, Sadeghi N, Hutcheson KA (2016). American Cancer Society Head and Neck Cancer Survivorship Care Guideline. CA Cancer J Clin.

[3] Epstein JB, Thariat J, Bensadoun RJ, Barasch A, Murphy BA, Kolnick L (2012). Oral complications of cancer and cancer therapy: from cancer treatment to survivorship. CA Cancer J Clin.

[4] Nekhlyudov L, Lacchetti C, Davis NB, Garvey TQ, Goldstein DP, Nunnink JC (2017). Head and Neck Cancer Survivorship Care Guideline: American Society of Clinical Oncology Clinical Practice Guideline Endorsement of the American Cancer Society Guideline. J Clin Oncol.

[5] Epstein JB, Stevenson-Moore P (2001). Periodontal disease and periodontal management in patients with cancer. Oral Oncol.

[6] Murphy BA, Deng J (2015). Advances in supportive care for late effects of head and neck cancer. J Clin Oncol.

[7] Bertl K, Loidl S, Kotowski U, Heiduschka G, Thurnher D, Stavropoulos A (2016). Oral health status and dental care behaviours of head and neck cancer patients: a cross-sectional study in an Austrian tertiary hospital. Clin Oral Investig.

[8] Kuhnt T, Stang A, Wienke A, Vordermark D, Schweyen R, Hey J (2016). Potencial risk factors for jaw osteoradionecrosis afterradiotherapy for head and neck cancer. Radiat Oncol.

[9] Schwarz E, Chiu GK, Leung WK (1999). Oral health status of southern Chinese following head and neck irradiation therapy for nasopharyngeal carcinoma. J Dent.

[10] Friedland PL, Bozic B, Dewar J, Kuan R, Meyer C, Phillips M (2011). Impact of multidisciplinary team management in head and neck cancer patients. Br J Cancer.

[11] World Health Organization (WHO) (1997). Oral Health Surveys: basic methods.

[12] Homann N, Tillonen J, Rintamäki H, Salaspuro M, Lindqvist C, Meurman JH (2001). Poor dental status increases acetaldehyde production from ethanol in saliva: a possible link to increased oral cancer risk among heavy drinkers. Oral Oncol.

[13] Chang JS, Lo HI, Wong TY, Huang CC, Lee WT, Tsai ST (2013). Investigating the association between oral hygiene and head and neck cancer. Oral Oncol.

[14] Divaris K, Olshan AF, Smith J, Bell ME, Weissler MC, Funkhouser WK (2010). Oral health and risk for head and neck squamous cell carcinoma: the Carolina Head and Neck Cancer Study. Cancer Causes Control.

[15] Deng J, Jackson L, Epstein JB, Migliorati CA, Murphy BA (2015). Dental demineralization and caries in patients with head and neck cancer. Oral Oncol.

[16] Gupta N, Pal M, Rawat S, Grewal MS, Garg H, Chauhan D (2015). Radiation-induced dental caries, prevention and treatment - A systematic review. Natl J Maxillofac Surg.

[17] Ammajan R, Joseph R, Rajeev R, Choudhary K, Vidhyadharan K (2013). Assessment of periodontal changes in patients undergoing radiotherapy for head and neck malignancy: a hospital-based study. J Cancer Res Ther.

[18] Jham BC, Reis PM, Miranda EL, Lopes RC, Carvalho AL, Scheper MA (2008). Oral health status of 207 head and neck cancer patients before, during and after radiotherapy. Clin Oral Investig.

[19] Madrid CC, Pauli Paglioni M, Line SR, Vasconcelos KG, Brandão TB, Lopes MA (2017). Structural Analysis of Enamel in Teeth from Head-and-Neck Cancer Patients Who Underwent Radiotherapy. Caries Res.

[20] Hong CH, Napeñas JJ, Hodgson BD, Stokman MA, Mathers-Stauffer V, Elting LS, Spijkervet FK, Brennan MT, Dental Disease Section, Oral Care Study Group, Multi-national Association of Supportive Care in Cancer (MASCC), International Society of Oral Oncology (ISOO) (2010). A systematic review of dental disease in patients undergoing cancer therapy. Supportive Care Cancer.

[21] Campos Velo MM, Farha AL, Silva Santos PS, Shiota A, Sansavino SZ, Souza AT (2017). Gamma radiation increases risk of radiation-related root dental caries. Oral Oncol.

[22] Dholam KP, Chouksey GC, Dugad J (2016). Oral health-related quality of life after prosthetic rehabilitation in patients with oral cancer: a longitudinal study with the Liverpool Oral Rehabilitation Questionnaire version 3 and Oral Health Impact Profile-14 questionnaire. Indian J Cancer.

[23] Schoen PJ, Raghoebar GM, Bouma J, Reintsema H, Vissink A, Sterk W (2007). Rehabilitation of oral function in head and neck cancer patients after radiotherapy with implant-retained dentures: effects of hyperbaric oxygen therapy. Oral Oncol.

[24] Finlay PM, Dawson F, Robertson AG, Soutar DS (1992). An evaluation of functional outcome after surgery and radiotherapy for intraoral cancer. Br J Oral Maxillofac Surg.

[25] Bascom PW (1968). Oral cancer and prosthodontics. J Prosthet Dent.

